# [Retracted] miR-217 inhibits the migration and invasion of HeLa cells through modulating MAPK1

**DOI:** 10.3892/ijmm.2025.5525

**Published:** 2025-03-31

**Authors:** Lihong Zhu, Shumei Yang, Jianfeng Wang

Int J Mol Med 44: 1824-1832, 2019; DOI: 10.3892/ijmm.2019.4328

Following the publication of this paper, it was drawn to the Editor's attention by a concerned reader that, regarding the Transwell invasion assay experiments shown in Figs. 2D and 5F, the 'Mimic control' panel in Fig. 2D appeared to contain an overlapping section of data with the 'Blank' data panel in Fig. 5F, such that data which were intended to show the results from differently performed experiments had apparently been derived from the same original source. In addition, the control western blot data (GAPDH protein bands) shown in the western blots in Figs. 3C and 5H were apparently the same, although the images had been inserted into these figures as mirror images of each other.

In view of the fact that these figures were assembled erroneously, the Editor of *International Journal of Molecular Medicine* has decided that this paper should be retracted from the Journal on account of a lack of confidence in the presented data. The authors were asked for an explanation to account for these concerns, but the Editorial Office did not receive a reply. The Editor apologizes to the readership for any inconvenience caused.

